# Carrier Redistribution Between Two Kinds of Localized States in the InGaN/GaN Quantum Wells Studied by Photoluminescence

**DOI:** 10.1186/s11671-019-2919-9

**Published:** 2019-03-12

**Authors:** Yao Xing, Degang Zhao, Desheng Jiang, Zongshun Liu, Jianjun Zhu, Ping Chen, Jing Yang, Feng Liang, Shuangtao Liu, Liqun Zhang

**Affiliations:** 1 State Key Laboratory of Integrated Optoelectronics, Institute of Semiconductors, Chinese Academy of Science, Beijing, 100083 China; 2 College of Materials Science and Opto-Electronic Technology, University of Chinese Academy of Sciences, Beijing, 100049 China; 3 Center of Materials Science and Optoelectronics Engineering, University of Chinese Academy of Sciences, Beijing, 100049 China; 4 Suzhou Institute of Nano-tech and Nano-bionics, Chinese Academy of Sciences, Suzhou, 215123 China

**Keywords:** Semiconductor materials, InGaN/GaN multiple quantum wells, Photoluminescence, Carrier localization

## Abstract

The InGaN/GaN multi-quantum wells (MQWs) are prepared at the same condition by metal-organic chemical vapor deposition (MOCVD) except the thickness of cap layers additionally grown on each InGaN well layer. The photoluminescence (PL) intensity of the thin cap layer sample is much stronger than that of thicker cap layer sample. Interestingly, the thick cap layer sample has two photoluminescence peaks under high excitation power, and the PL peak energy-temperature curves show an anomalous transition from reversed V-shaped to regular S-shaped with increasing excitation power. Meanwhile, it exhibits a poorer thermal stability of thick cap layer sample under higher excitation power than that under lower excitation power. Such an untypical phenomenon is attributed to carrier redistribution between the two kinds of localized states which is induced by the inhomogeneous distribution of indium composition in thick cap layer sample. Furthermore, the luminescence of deep localized states has a better thermal stability, and the luminescence of shallow localized states has a poor thermal stability. In fact, such a severer inhomogeneous indium distribution may be caused by the degradation of subsequent epitaxial growth of InGaN/GaN MQWs region due to longer low-temperature GaN cap layer growth time.

## Introduction

InGaN/GaN multi-quantum well (MQW) structure has received great attention due to its wide use in light-emitting diodes (LEDs) and laser diodes (LDs) [1–6]. Although the high threading dislocation density and reduction of wave function overlap caused by spontaneous and piezoelectric polarization of InGaN/GaN MQWs, their luminance efficiency is still surprisingly high [7–10]. One of the main reasons is that the localization of excitons in potential minima due to fluctuations of indium content leads to the formation of quantum-dot-like states in InGaN/GaN quantum wells [11]. However, it remains ambiguous how the roles localization states play on the luminescence mechanism. Several studies have reported the effect of InGaN composition fluctuations on radiative and Auger recombinations [12–14]. Theoretical simulations of atomistic tight-binding used by Jones found that the localization increases both radiative and Auger recombination rates, but Auger recombination rate increases by one order of magnitude higher than radiative one [15]. Experimentally, carrier localization leads to the relaxation of the k-selection rule in the Auger recombination process, and thus strongly enhances the Auger recombination process in polar InGaN/GaN QWs under high optical excitation [16]. It is well known that the temperature-dependent S-shaped behavior of luminescence peak energy is a fingerprint of carrier localization. Many models, such as localized state ensemble (LSE) model, are proposed to explain the carrier localization and thermal redistribution behavior, showing that the variation of luminescence peak energy with temperature can be influenced by unique carrier redistribution process under different excitation levels [17–21]. Generally, the manufactured devices like laser diodes are always operating with a higher injected carrier density [22]. In this case, the photoluminescence spectra of localized states may exhibit a unique behavior at different excitation level associated with the uniformity of localized states. Further studies are therefore necessary to understand the effect of alloy fluctuations on InGaN devices.

In this work, two typical samples with different thickness of GaN cap layers which are additionally grown on each InGaN well layer are prepared by metal-organic chemical vapor deposition system (MOCVD). The properties of MQWs are characterized in detail by high-resolution X-ray diffraction (HRXRD), temperature-dependent photoluminescence (TDPL), and power-dependent photoluminescence (PDPL) measurements. It is found that the thick cap layer sample shows an anomalous peak at higher energy side under high optical excitation power. This implies a co-existence of two different kinds of localized states. Meanwhile, the PL intensity decays faster at low temperatures when the excitation power is increasing higher. Hence, we can assume that the photoluminescence of deep localized states has a better thermal stability, and the photoluminescence of shallow localized states has a poor thermal stability.

## Methods

### Materials

**Fig. 1:**
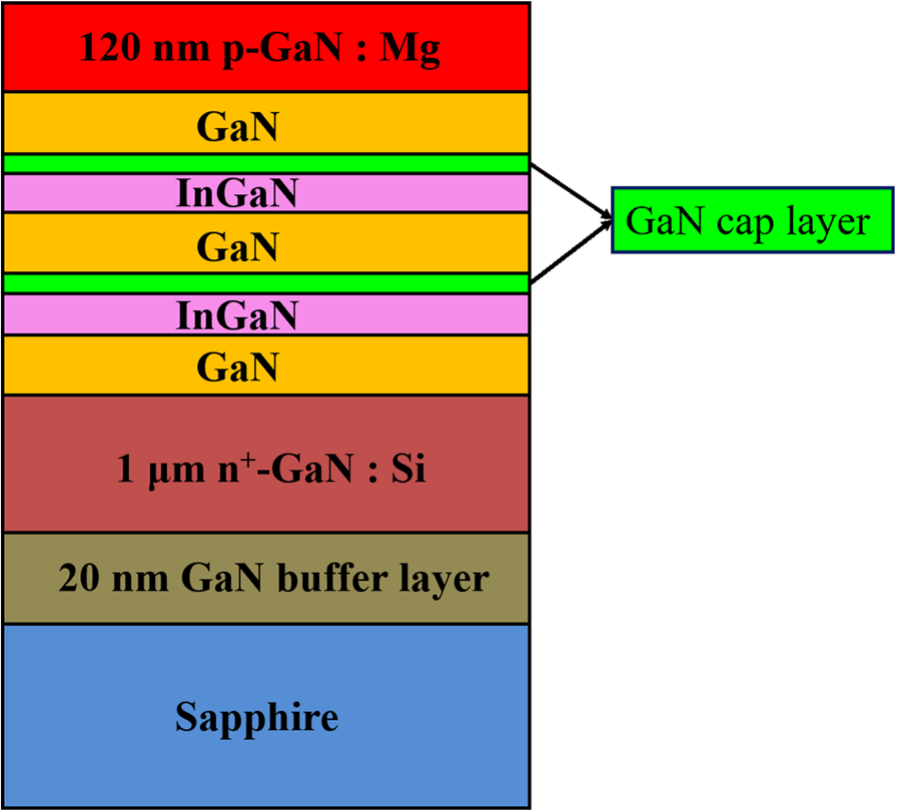
The cross-sectional schematic diagram of the epilayer structures of two MQWs

The InGaN/GaN MQW samples with different cap layer thickness grown on c-plane sapphire substrate by an AIXTRON 3 × 2 in close-coupled showerhead reactor are studied. Trimethylgallium (TMGa), trimethylindium (TMIn), and ammonia (NH_3_) were used for the epitaxial growth as Ga, In, and N source precursors, respectively, in which H_2_ and N_2_ were the carrier gas of the GaN and InGaN growth, respectively. MQW is consisted of two periods of InGaN/GaN QWs. During the growth of each well layer, the TMIn flow rate was kept constant. Then a GaN cap layer was grown at the same temperature as well layer, i.e., 710 °C. Afterwards, the temperature was ramped up to 830 °C, and stay several seconds, and then the barrier layer was grown at 830 °C. Both samples A and B are grown under the same conditions except the GaN cap layer growth time, i.e., it is 30 s for sample A and 200 s for sample B. The schematic diagram of structure and growth parameters of two InGaN/GaN MQWs A and B are shown in Fig. 1.

### Characterization

To determine the average indium content, period thickness, and material quality of two InGaN/GaN MQWs, high-resolution x-ray diffraction (HRXRD) measurement is performed with Rigaku Ultima IV with Cu-Ka radiation (λ = 1.54 Å) that operated at 40 kV and 30 mA. For temperature-dependent photoluminescence (TDPL) and excitation power-dependent PL (PDPL) measurements, a 405 nm laser was used as an excitation light source with spot size of 0.5 mm^2^, and the excitation power varied from 0.01 to 50 mW. The samples were mounted in a closed-cycle He cryostat and the temperature was controlled from 10 to 300 K.

## Results and Discussions

**Fig. 2:**
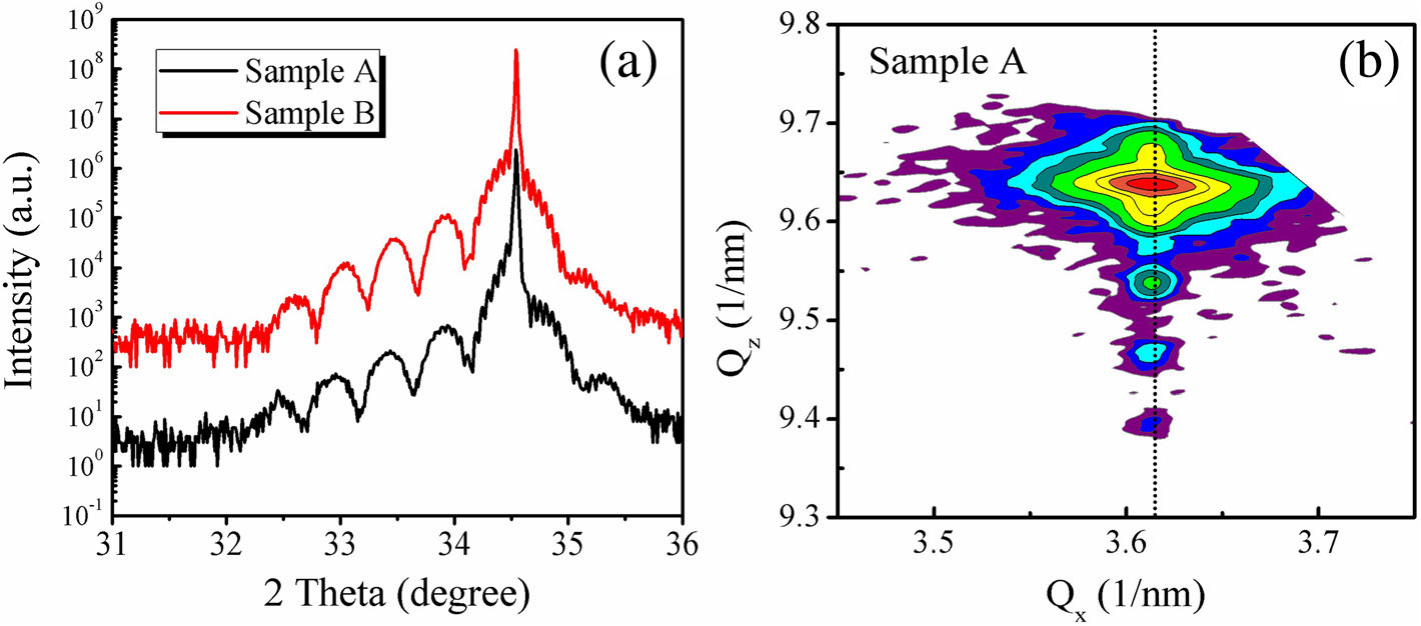
Omega-2theta scans and reciprocal space mapping of both samples are performed by HRXRD. **a** HRXRD Omega-2theta curves on GaN (0002) plane for samples A and B. **b** Reciprocal space mapping (RSM) for the GaN (10–15) diffraction of sample A

To investigate the structural properties of two samples A and B, the ω-2θ symmetrical (0002) scans have been carried out, as shown in Fig. 2a. The substrate peak originates from GaN (002) plane, and satellite peaks come from MQWs. Satellite peaks up to the fourth order can be clearly observed in all two samples, indicating a good layer periodicity. In addition, the average indium composition and periodic thickness can be obtained by fitting the measured curves, as shown in Table 1. It can be seen that as the thickness of cap layer increases, the GaN barrier thickness and the thickness and indium composition of InGaN well layers increase slightly. Actually, because the growth rate of cap layer is as small as 0.006 nm/s and the growth temperature is as low as 710 K, the change of barrier thickness is relatively small. However, noting that the growth of additional GaN cap layers may have influences not only on the barrier layer thickness but also on the diffusion, evaporation, and redistribution of In atoms in the InGaN well layers, as will be discussed in detail later.

**Table 1:** Structural parameters of InGaN/GaN MQWs of samples A and B determined by HRXRD

Samples	Cap layer growth time (s)	Period thickness (nm)	Barrier thickness (nm)	Well thickness (nm)	In content in InGaN
A	30	19.10	14.85	4.25	10.0%
B	200	19.95	15.50	4.45	10.3%

Meanwhile, to examine the strain state of GaN QB and InGaN QW layers, reciprocal space mapping (RSM) in the vicinity of the GaN (10–15) plane is performed. The result of sample A is shown in Fig. 2b (The RSM figure of B is similar, but not shown here). We can observe that for sample A, the satellite peaks of MQW and GaN peak align well on the same vertical line, indicating that the MQWs of both samples are fully strained without any relaxation [23].

**Fig. 3:**
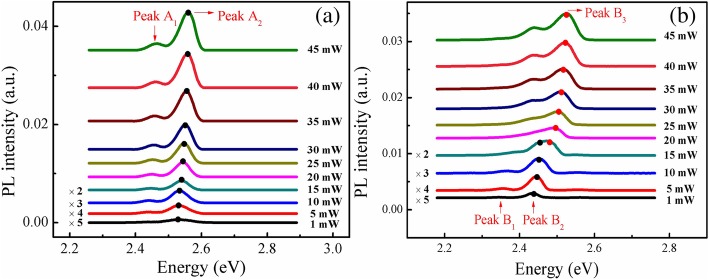
PL spectra of samples A (**a**) and B (**b**) at several different excitation powers, measured under temperatures of 10 K

Figure 3 shows the PDPL measurements for two samples at 10 K. It is interesting to find that the two samples exhibit quite different behaviors. For sample A, there is a small peak located at lower energy side (peak *A*_1_) of dominant peak *A*_2_. It is confirmed that peak *A*_1_ is phonon replica which is 92 meV away from the main peak *A*_2_. The phonon replica of *B*_2_ also appears in sample B and is called as peak *B*_1_. On the other side, in Fig. 3b, it can be observed that there is only one dominant luminesce peak *B*_2_ with the excitation power lower than 5 mW. However, when the excitation power is higher than 10 mW, another peak *B*_3_ abnormally appears at higher energy side of *B*_2_, and peak *B*_3_ gradually becomes the dominant emission peak instead of peak *B*_2_ when the excitation power increases further. Here, we can assume that the majority of the optical excited carriers first got trapped in the first type of electronic states (e.g., localized states created by the local In-rich clusters), and then radiatively recombine, producing luminescence peak *A*_2_ and *B*_2_. [24].

**Fig. 4:**
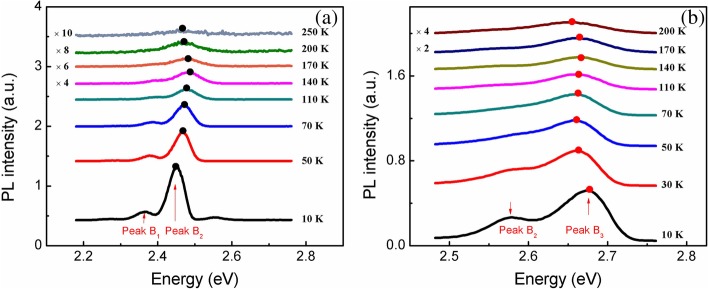
PL spectra of sample B at a temperature range of 10–300 K, measured under excitation powers of 5 mW (**a**) and 40 mW (**b**)

To examine the behavior of anomalous peak *B*_3_ of sample B further, we have performed the TDPL measurements under different excitation powers shown in Fig. 4, in which Fig. 4a and b are the PL spectra obtained under excitation power of 5 mW and 40 mW, respectively. Note that the two-peak phenomenon of the emission spectra in Fig. 4b was clearly seen at temperatures below 200 K and became blurred toward 300 K. Summarizing the emission spectra behavior, one can see that the transition from the low energy emission peak to the high energy emission peak occurs in a narrow range of excitation power and has a “switching” character. Outside the narrow transition region, single low energy (*B*_2_) or high energy (*B*_3_) emission peak dominates at low and high excitation powers, respectively.

**Fig. 5:**
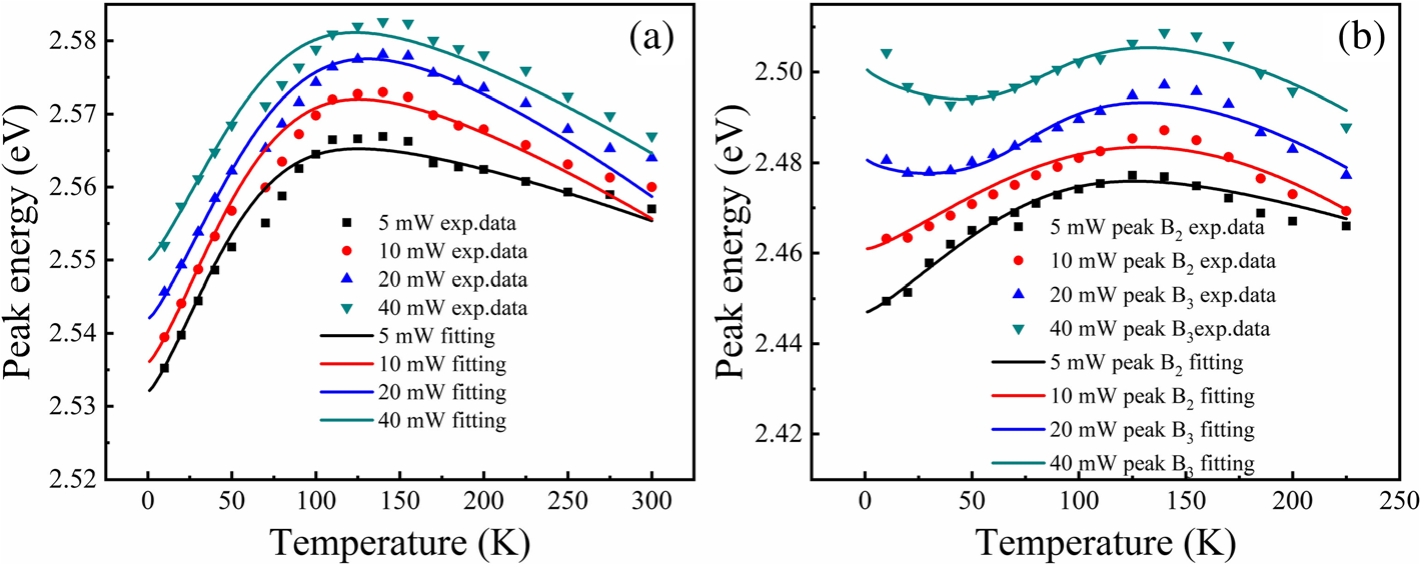
PL emission peak energy as a function of temperature for samples A (**a**) and B (**b**) under different excitation powers. The solid lines are theoretical fitting curves using LSE model. The dots are the experiment data

Additionally, a closer inspection of the variation of dominant emission peak energy with temperature of both samples, we can find something unique. As shown in Fig. 5a, when the excitation power increases from 5 to 40 mW for sample A, the variation of PL peak energy with increasing temperature (called E-T curve below) shows “reversed V shape” curves, which is different from the regular “S” shape. The reversed V shape is almost unchanged with increasing excitation power except an overall blue shift of the peak energy. The reversed “V”-shaped temperature dependence is explained as a joint action of carrier filling effect at luminesce centers and bandgap shrinkage effect accompanied with increasing temperature [25, 26]. On the other hand, as shown in Fig. 5b, the E-T curves for sample B under the excitation power lower than 5 mW show a reversed V shape. This situation is similar to sample A. However, when the excitation power gradually increases to 40 mW, a first redshift appears at lower temperature range, and the E-T curves have a regular S shape. Apparently, this phenomenon contradicts the expectation that when the excitation power is large enough, the localization effect will completely disappear, and the temperature behavior of the peak energy will closely follow the Varshni law [27].

Thus, to quantitatively explain the observed anomalous excited optical power dependence of luminescence of localized states, the LSE luminescence model was employed to fit the E-T curves, which is proposed by Q. Li et al. This model can be used in all temperature ranges, and it can not only fit the “S” shape E-T curves but also the “V” or reversed “V” shapes. In addition, it was also proved that LSE model can be reduced to Eliseev et al.’s band-tail model at high temperatures [24, 25]. In this model, the peak energy as a function of temperature can be described as [18–21]:

1 $$ E(T)=\left({E}_0-\frac{\alpha {T}^2}{\theta +T}\right)-{xk}_BT $$

where *θ* is the Debye temperature of the specific material and *a* is the Varshni parameter, *k*_B_ is the Boltzmann constant, and *x* can be numerically solved by the following transcendental equation [18–21]:

2 $$ {xe}^x=\left[{\left(\frac{\sigma }{k_BT}\right)}^2-x\right]\left(\frac{\tau_r}{\tau_{tr}}\right){e}^{\left({E}_0-{E}_a/{k}_BT\right)} $$

where *σ* is the standard deviation of distribution of the localized states. In other words, it means the width of Gaussian-type state density distribution. *τ*_*r*_ and *τ*_*tr*_ represent the radiative recombination and escape lifetime of the localized carrier, and thus *τ*_*r*_/*τ*_*tr*_ implies the portion of carriers which recombines nonradiatively. *E*_0_ is the central energy of the localized centers, and *E*_a_ gives a “marking” level below which all localized states are occupied by carriers at 0 K which is just like the quasi-Fermi level in the Fermi-Dirac distribution. It is obvious that *E*_0_ and *E*_a_ together are related to the origin of luminescence localized centers [17].

The obtained fitting parameters of both samples are shown in Table 2. For sample A, the central energy *E*_0_ and *E*_a_ changes to 19 meV and 18 meV from 5 to 40 mW, respectively. It is noticed that the *E*_0_-*E*_a_ and *σ* is almost unchanged. It is because that as the excited power increases, more and more carriers will be excited. First, the strong piezoelectric field of InGaN wells will be screened by the photogenerated carriers, leading to an increase of central energy *E*_0_. Second, more and more carriers will occupy higher electronic states according to the filling effect, which results in the increase of quasi-Fermi level of localized carriers *E*_a_. Therefore, *E*_0_-*E*_a_ represents the joint action of polarization screening effect and carrier filling effect, and thus an overall blueshift in peak position for sample A is observed. Unlike sample A, for sample B, from 5 to 40 mW, there are bigger increases of *E*_0_ and *E*_a_, 73 meV and 57 meV, respectively. *E*_0_-*E*_a_ increase by 16 meV, *τ*_*r*_/*τ*_*tr*_ changes by several orders of magnitude, and *σ* decreases a little. Such changes are so big that we have to assume that the origin of luminescence centers is different at different excitation powers of 5 mW and 40 mW.

**Table 2:** Fitting parameter in the LSE model for two samples

Samples	Excited power (mW)	*E*_0_ (eV)	*E*_a_ (eV)	*E*_0_-*E*_a_ (meV)	$\frac{\tau_r}{\tau_{tr}}$	*σ* (meV)
Sample A	5	2.57	2.532	38	0.004	14
	10	2.579	2.538	41	0.004	14
	20	2.585	2.545	40	0.004	15
	40	2.589	2.55	39	0.003	15
Sample B	5	2.486	2.447	39	0.003	24
	10	2.516	2.461	55	0.009	30
	20	2.541	2.481	60	1.99	19
	40	2.559	2.504	55	15.01	20

**Fig. 6:**
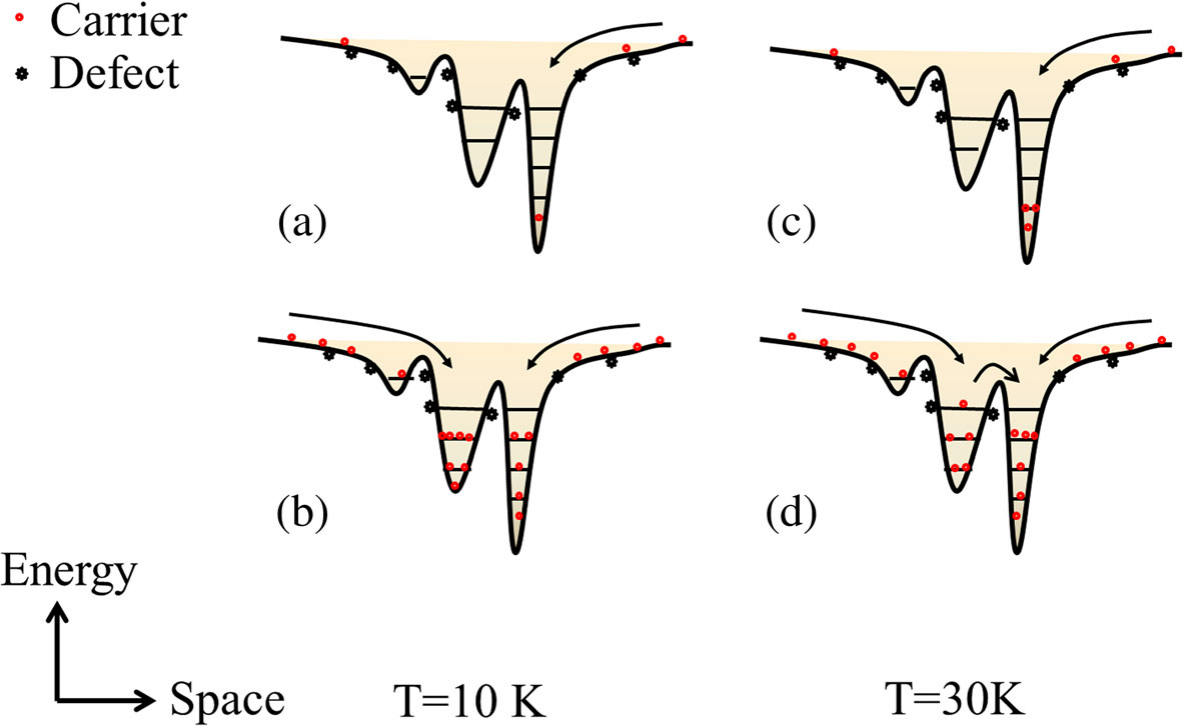
Schematic diagrams indicating the possible mechanism of the anomalous variation of the PL peak energy vs. T curves with different excitation powers. The carrier distributions at lower T (10 K) are shown in (**a**) and (**b**) for *P* = 5 mW and 40 mW, respectively. The carrier distributions at higher T (30 K) are shown in (**c**) and (**d**) for *P* = 5 and 40 mW, respectively

Therefore, it is suggested that for sample B, there are two kinds of localization states, distributing at two different energy depths in the well layers due to the inhomogeneous distribution of indium composition, i.e., with higher indium composition (deep localization states) and lower indium composition (shallow localization states). In addition, to explain the above phenomenon of sample B, the schematic diagrams indicating the possible mechanism of the carrier redistribution between two kinds of localized states are plotted in Fig. 6. At 10 K, under lower excitation power such as 5 mW, shown in Fig. 6a, the majority of the optical excited carriers first get trapped in the first type of electronic states (deep localized states) and thus the lower energy peak dominates, while at 40 mW, shown in Fig. 6b, more and more photogenerated carriers will occupy the higher energy level, and then the shallow localized states with the higher energy state density are also filled, and thus the higher energy peak dominates gradually with the increase of excited power. Therefore, *E*_*0*_ and *E*_a_ increase a lot, and *τ*_*r*_/*τ*_*tr*_ increase by several orders which imply the escape ability of carriers out of localized states. As the temperature increases to 30 K, at 5 mW, as shown in Fig. 6c, the photogenerated carriers with a certain amount of thermal energy are mainly used to filling the deeper localized states, resulting in a first blueshift of E-T curves; however, in Fig. 6d, when it comes to 40 mW, based on the assumption that shallow localized states have more capacity than the deep localized states, the majority of the photogenerated carriers stay in shallow localized states, and it will be able to transfer to deep localized states which has a strong ability to bind carriers. Therefore, the E-T curves redshift. In other words, the appearance abnormal changes of E-T curves are concerned with multiple kinds of localization states due to the inhomogeneous indium distribution in InGaN well layers of sample B. And such compositional fluctuations are supposed to be mainly due to the random alloy fluctuations on an atomic scale [28].

**Fig. 7:**
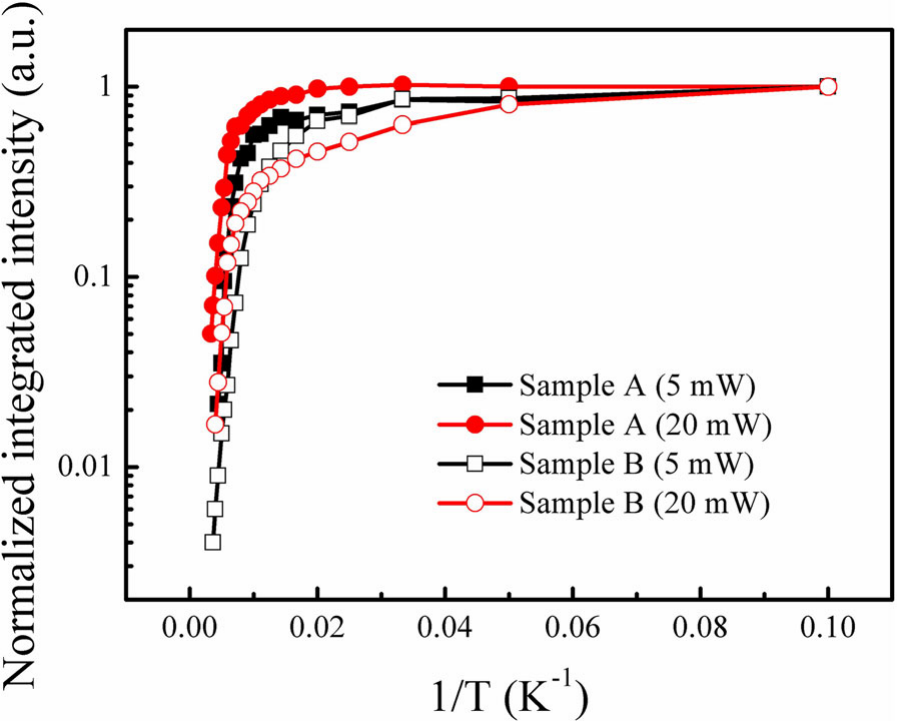
The integrated intensity extracted from PL spectra of both samples at a temperature range of 10–300 K, measured under excitation powers of 5 mW and 20 mW

Furthermore, the appearance of high energy emission peak under high excitation power of sample B also leads to an anomalous variation of the PL integrated intensity. In Fig. 7, the integrated intensity vs. temperature curves of samples A and B measured at excitation powers of 5 mW and 20 mW are plotted, respectively. First, note that the thermal quenching of sample B is obviously quicker than that of sample A. Generally, the luminescence thermal quenching of the InGaN MQWs is dominated by the nonradiative recombination processes which can be described by Arrhenius equation. Therefore, the quick thermal quenching implies a poor thermal stability of sample B. Furthermore, when the excitation power is high enough, the impact of nonradiative recombination centers at relatively low temperatures will not be so much significant, because the nonradiative recombination centers are easily saturated by excess carriers [27]. This can perfectly explain the slower variation of PL integrated intensity vs. 1/T curves with the increase of excitation power of sample A. However, it is quite interesting for sample B that the normalized integrated intensity under excitation power of 5 mW is even higher than that under 20 mW when the temperature is lower than 125 K, and it turns to be opposite at temperatures higher than 125 K. As mentioned before, it is assumed that only one lower energy emission peak which is originated from deep localized states is dominant at 5 mW, while another higher one originated from shallow localized states becomes dominant at 20 mW. Therefore, it is concluded that the deep localized luminescence centers have better luminescence efficiency than shallow luminescence centers. This agrees well with the previous research result related to the localized states [28]. Therefore, it can also be proved, to some extent, that there are two kinds of localized states excited at 20 mW for sample B.

Based on all these analyses, we demonstrate that peak *B*_3_ originates from the localization states with lower potential related to the inhomogeneous distribution of indium composition of sample B. It agrees well with the experimental results of the higher emission energy peak *B*_3_ and the reduction of IQE of sample B at low temperatures under higher excitation power. Actually, in the growth process of QWs, considering the pulling effects, indium atoms are prone to accumulate at the top of InGaN QW layer and form an extra layer known as indium floating layer [29]. Thicker GaN cap layer growth at low temperature is detrimental to the evaporation of these indium floating atoms. Consequently, In atoms may incorporate into GaN cap layer and barrier layer after the QW growth [30]. Naturally, this behavior will result in an increase of well layer thickness, and thus the QCSE is enhanced. The higher strain and the stronger piezoelectric field in the active QW would induce more localized relaxation and, thus, deeper localized potentials and higher barriers. Meanwhile, more dislocations and defects are introduced into the subsequent growth of GaN barrier layer. Generally, there is large tensile stress near dislocations, and indium atoms may tend to accumulate near the dislocations and distribute inhomogeneously. [31, 32] Therefore, in the growth of InGaN well layer, there are more indium-rich and indium-poor areas associated with the increasing dislocation density. It means that the scale of indium fluctuations will become larger as the capping layer thickness increases. In our experiments, it shows that two different kinds of localization states are introduced into sample B with thicker cap layer, and the PL peak of higher emission energy is activated under higher excitation power. On the other hand, the photogenerated carrier stay at deep localized states can screen away the defects and thus has a better thermal stability, while the photogenerated carrier stay at shallow localized states will be captured by the defect-related nonradiative recombination once they can overcome the relatively lower barrier height.

## Conclusions

In summary, the InGaN/GaN multi-quantum well (MQW) samples with different thickness of GaN cap layers additionally grown on the InGaN well layers are prepared by metal-organic chemical vapor deposition system (MOCVD). Their structural and optical properties are investigated by HRXRD, TDPL, and PDPL measurements and analyzed. PDPL results show that an additional high-emission energy peak is excited at higher excitation power only for sample B which is grown with thick cap layers. Meanwhile, TDPL results measured at different excitation powers for sample B reveal that the E-T curves of dominant PL peak change from reversed V shape to regular S shape when the excitation power increases. Additionally, a poorer thermal stability of sample B at high excitation power was found. These anomalous phenomena imply that there are two kinds of localized states of sample B which are induced by the relatively inhomogeneous indium distribution. These conclusions give us a further understanding of photoluminescence mechanism of green InGaN/GaN quantum wells and inhomogeneity effect at high excitation level which may help us in the manufacturing of InGaN/GaN laser diodes.

## Abbreviations

HRXRD: High-resolution X-ray diffraction
LDs: Laser diodes
LEDs: Light-emitting diodes
LSE: Localized state ensemble
MOCVD: Metal-organic chemical vapor deposition system
MQWs: Multi-quantum wells
NH_3_: Ammonia
PDPL: Power-dependent photoluminescence
RSM: Reciprocal space mapping
TDPL: Temperature-dependent photoluminescence
TMGa: Trimethylgallium
TMIn: Trimethylindium
